# Enteric methane research and mitigation strategies for pastoral-based beef cattle production systems

**DOI:** 10.3389/fvets.2022.958340

**Published:** 2022-12-23

**Authors:** Paul E. Smith, Alan K. Kelly, David A. Kenny, Sinéad M. Waters

**Affiliations:** ^1^Teagasc, Animal and Bioscience Research Department, Animal and Grassland Research and Innovation Centre, Dunsany, Ireland; ^2^UCD School of Agriculture and Food Science, University College Dublin, Dublin, Ireland

**Keywords:** methane, beef cattle, rumen microbiome, pasture, nutrition, breeding

## Abstract

Ruminant livestock play a key role in global society through the conversion of lignocellulolytic plant matter into high-quality sources of protein for human consumption. However, as a consequence of the digestive physiology of ruminant species, methane (CH_4_), which originates as a byproduct of enteric fermentation, is accountable for 40% of global agriculture's carbon footprint and ~6% of global greenhouse gas (GHG) emissions. Therefore, meeting the increasing demand for animal protein associated with a growing global population while reducing the GHG intensity of ruminant production will be a challenge for both the livestock industry and the research community. In recent decades, numerous strategies have been identified as having the potential to reduce the methanogenic output of livestock. Dietary supplementation with antimethanogenic compounds, targeting members of the rumen methanogen community and/or suppressing the availability of methanogenesis substrates (mainly H_2_ and CO_2_), may have the potential to reduce the methanogenic output of housed livestock. However, reducing the environmental impact of pasture-based beef cattle may be a challenge, but it can be achieved by enhancing the nutritional quality of grazed forage in an effort to improve animal growth rates and ultimately reduce lifetime emissions. In addition, the genetic selection of low-CH_4_-emitting and/or faster-growing animals will likely benefit all beef cattle production systems by reducing the methanogenic potential of future generations of livestock. Similarly, the development of other mitigation technologies requiring minimal intervention and labor for their application, such as anti-methanogen vaccines, would likely appeal to livestock producers, with high uptake among farmers if proven effective. Therefore, the objective of this review is to give a detailed overview of the CH_4_ mitigation solutions, both currently available and under development, for temperate pasture-based beef cattle production systems. A description of ruminal methanogenesis and the technologies used to estimate enteric emissions at pastures are also presented.

## Introduction

The global population is expected to exceed nine billion people by the year 2050 (1). To meet the nutritional requirements of the growing population, global agricultural output will be required to increase by an estimated 50% (2). At present, agriculture is accountable for 10–12% of global anthropogenic greenhouse gas (GHG) emissions (3), with emissions from the sector likely to rise in response to increased agricultural output. In light of the urgent need to decrease anthropogenic GHG emissions to limit global surface temperature increase (4), mitigating the environmental burden associated with a rise in global agricultural output will be a challenge.

Global food production has become reliant on ruminant livestock due to the ability of ruminants to transform inaccessible energy stored in plants into high-quality sources of protein and energy for human consumption (5). However, methane (CH_4_), a potent GHG, estimated to have 28 times greater warming potential than carbon dioxide (CO_2_) (6), is produced as a metabolic byproduct of the ruminal fermentation of feed by the rumen microbial community (7). Indeed, CH_4_ originating from enteric fermentation accounts for the majority of global agriculture's 36.5% contribution to anthropogenic CH_4_ emissions (8). Over the past two decades, numerous CH_4_ abatement strategies have been developed for ruminant livestock (9–12); however, not all strategies will be easily implemented at the farm level, in particular in pasture-based production systems. The development of anti-methanogenic vaccines would likely appeal to livestock producers; however, the duration of their effectiveness has proven to be an obstacle to their implementation (13). Dietary supplementation may have a role when cattle are housed. However, the supplementation of beef cattle at pasture is likely to be less feasible (14), but may be implemented as part of a winter housing period or as part of a supplementary feeding regime at pasture. Subsequently, due to the low profitability of beef cattle production (15), any CH_4_ supplementation strategy would at minimum need to be cost-neutral to ensure uptake at the farm level. Mitigation strategies which focus on improving the growth rate of beef cattle at pasture will be advantageous if they reduce the lifetime emissions of growing cattle. However, the suckler cow herd can account for ~80% of the enteric CH_4_ produced within a cow-calf production system (16). As a result, strategies that can lower the daily CH_4_ output of an animal will also be required to improve the environmental sustainability of the sector. Equally, animal breeding has been advocated by numerous authors as a mitigation strategy due to its ability to deliver permanent GHG reductions to future generations of livestock (17, 18).

This review aims to give an overview of the relationship of the rumen microbiota with methanogenesis and research focused on developing CH_4_ mitigation strategies for pasture-based beef cattle production systems. For dietary-focused mitigation strategies, an emphasis is placed on those which are likely to be practically feasible for a temperate pasture-based production system similar to that of the Irish and New Zealand beef cattle production systems. The benefits and limitations on enteric CH_4_ emissions and animal productivity are discussed for anti-methanogenic vaccinations, pasture management, dietary supplementation, and animal selection-based strategies. Due to potential impacts on animal health and lack of data on seaweeds sourced from temperate geographic regions, the CH_4_ mitigation potential of supplementing nitrate and seaweeds has not been included but has been reviewed by others (9–12, 19). In addition, while this review will predominantly focus on research conducted in beef cattle, reference to data originating from other ruminants will be utilized where evidence in beef animals is lacking.

## Enteric methane

In the rumen, bacteria, protozoa, and fungi hydrolyze plant polysaccharides to monomeric sugars, which are further fermented to produce various products, such as volatile fatty acids (VFA) acetate, propionate, and butyrate along with CO_2_ and CH_4_ (20). The Embden–Meyerhof–Parnas (EMP) pathway is the initial pathway in the catabolism of sugars, which results in the formation of pyruvate, a central intermediary metabolite in the rumen (21). During the EMP pathway, carbohydrates are oxidized resulting in the reduction of the electron transporter nicotinamide adenine dinucleotide (NAD+) to NADH, which must be subsequently reoxidized to NAD+ for further fermentation to continue (13). An excessive amount of dihydrogen (H_2_) in the rumen has the ability to inhibit the activity of hydrogenase enzymes, therefore, limiting the oxidation of sugars when alternative pathways for H_2_ disposal are absent (13). Methanogenesis contributes to the efficiency of the rumen, preventing increases in the partial pressure of H_2_ and therefore promoting the function of microbial enzymes involved in electron transfer reactions, such as NADH dehydrogenase (22). The rumen provides a unique environment characterized by a relatively rapid passage rate and a readily available supply of CO_2_ and H_2_, resulting in a community of archaea distinct from other anoxic systems (23). During the dominant methanogenesis pathway (hydrogenotrophic methanogenesis) conducted by rumen methanogens, H_2_ is oxidized to H+, and CO_2_ is reduced to form CH_4_ (24). However, CH_4_ can also be produced *via* the reduction of methyl compounds and acetate *via* methylotrophic and acetoclastic methanogenesis, respectively (Table 1). Methanogenesis is considered an energy-inefficient process, which consumes an estimated 2–12% of the animal's gross energy intake (GEI) (26, 27). After its synthesis in the rumen, the bulk of enteric CH_4_ is expelled from the rumen *via* eructation (28).

**Table 1:** The three main methanogenesis pathways in the rumen with estimates of free energies from reactions.

**Pathway**	**Formula**
Hydrogenotrophic methanogenesis^a^	CO_2_ + 4H_2_ → CH_4_ + 2H_2_O
Methylotrophic methanogenesis^b^	CH_3_OH +H_2_ → CH_4_ + H_2_O
Acetoclastic methanogenesis	CH_3_COOH → CH_4_ + CO_2_

Table sourced from Waters et al. (5) and originally modified from Liu and Whitman (25) and Ferry (24). The reader is directed toward Liu and Whitman (25) and Ferry (24) for a more in-depth discussion on the biochemistry of methylotrophic and acetoclastic methanogenesis.

a Formate is also capable of being used, however, not included separately due to its conversion to CO_2_ prior to being utilized.

b Most thermodynamically favorable methyl-based reaction.

### Hydrogenotrophic methanogenesis pathway

In nature, three major substrates are used in the production of CH_4_: CO_2_, compounds containing methyl groups, and acetate (25). In the hydrogenotrophic pathway, CH_4_ is produced from the reduction of CO_2_ with electrons donated from H_2_ and formate (25, 29). Compounds containing methyl groups also provide a carbon source for CH_4_ production (30). In the acetoclastic pathway, the carbonyl group of acetate is cleaved and oxidized to CO_2_ (24).

Hydrogenotrophic methanogenesis is a seven-step process, which results in the carbon group progressing through the pathway *via* attachment to various coenzymes and finally getting reduced to CH_4_ (31). The pathway commences with the binding of CO_2_ with methanofuran (MF) and the reduction of CO_2_ to a formyl group with ferredoxin (Fd) acting as the electron donor (25) and formyl-methanofuran dehydrogenase (fmd) as the catalyst for the reaction (24). In the second step of the pathway, the formyl group of the formyl–MF complex is transferred to tetryahydromethanopterin (H_4_MPT) (32). In step three, the formyl group of formyl–H_4_MPT is converted to methenyl–H_4_MPT by the enzyme 5,10-methenyltetrahydromethanpterin cyclohydrolase (31). As part of steps four and five of the pathway, coenzyme F_420_ (F_420_ H_2_) acts as the electron donor on two occasions, firstly converting the methenyl group to a methylene group (step four) followed by a further reduction to a methyl group (step five) resulting in the formation of methyl–H_4_MPT (25). The oxidation of F_420_ is catalyzed by F_420_-reducing hydrogenase (33). In the sixth step, the methyl group is transferred to coenzyme M (HS-CoM), with the formation of methyl-CoM catalyzed by CH_3_-H_4_MPT:coenzyme M methyltransferase (*mcr*) (24). The final reduction step, catalyzed by Methyl-coenzyme M reductase *(mcr)*, reduces methyl-CoM to CH_4_ (25). Coenzyme B (HS-CoB) provides the electrons for the final step of the pathway and combines with the cleaved HS-CoM forming the heterodisulfide molecule CoM-S-S-CoB as the end product (24) along with CH_4_.

The hetrosulfide end product, CoM-S-S-CoB, is returned to the reduced molecules HS-CoB and HS-CoM *via* the cytosol-encompassed hydrogenase–heterodisulfide reductase complex (MvhADG–HdrABC) (34). Electron bifurcation of H_2_ results in the reduced products HS-CoB and HS-CoM and a reduction of ferredoxin for the methanogenesis cycle (35). A further supply of reduced ferredoxin is created from the energy produced during the translocation of sodium (Na^+^) ions into the cell *via* membrane-bound hydrogenases, Eha and Ehb, oxidizing H_2_ (34, 35).

### Expressions of the methanogenic output of ruminant livestock

The methanogenic output of an animal is predominantly reported in four different ways. Enteric CH_4_ emissions, reported in grams (g/day) or liters (l/day), can be expressed as daily CH_4_ emissions (DME). However, DME is largely influenced by an animal's voluntary feed intake, as the quantity of feed an animal consumes will influence the availability of substrate in the rumen for fermentation. Ratio expressions, relative to feed intake, had previously been advocated to overcome the influence of dry matter intake (DMI) on DME (36). Enteric emissions can therefore be expressed as a proportion of feed intake, known as CH_4_ yield (MY; g/kg of DMI). Enteric emissions can also be expressed per unit of animal product produced, which is referred to as CH_4_ intensity (MI; g/kg of milk yield or carcass weight). In growing animals, MI is also reported per unit of average daily gain (MADG; g/kg of ADG), an indicator of the environmental efficiency of animal growth. More recently, the concept of residual CH_4_ emissions (RME; g/day) has been proposed by Herd et al. (37) and can be defined as the difference in the animal's actual and expected CH_4_ output, based on its level of feed intake and body weight (38). The RME index was initially derived from the linear regression of DME on DMI (37); however, more recently, it was calculated as the multiple linear regression of DME on DMI and body weight (38).

### Sources of variation in enteric methane emissions

Daily CH_4_ emissions are a variable trait with coefficients of variation (CV; %) of 12.7–19.2% reported in large populations of beef cattle (36, 38). However, the underlying biological mechanisms contributing to inter-animal divergence in enteric emissions are not yet fully understood, although factors including host genetics, voluntary feed intake, dietary composition, the rumen microbiome, and digestive tract physiology are likely influencers. Furthermore, the methodology used to estimate enteric emissions may also influence our interpretation of the methanogenic output of an animal.

Methane output is a heritable trait indicating that it is under some degree of host control with heritability estimates ranging from 0.19 in Danish Holstein cows [*n* = 750; (39)] to 0.30 in Australian Angus cattle [*n* = 1,060; (36)] reported for DME. The heritability of MY, MI, and RME has been estimated as 0.20, 0.25, and 0.19 in beef cattle (40) and ranged from 0.23 to 0.30, 0.33 to 0.42, and 0.18 in dairy cows (40, 41), respectively. In addition, in some studies, breed has been shown to have a significant impact on DME (42, 43). However, when emissions were reported as MY in the previous studies, the effect of the breed was insignificant, indicating differences in DME were likely influenced by inter-animal variation in DMI. Indeed, DME has been shown to be both genetically and phenotypically correlated with DMI (38, 40, 44) with up to 85 and 86% of the variation in DME explained by DMI based on the meta-analysis of published literature by Ramin and Huhtanen (45) and Hristov et al. (46), respectively. Intuitively, the relationship of DME with DMI is to be expected as a greater volume of feed enters the rumen, there will be an increase in ruminal fermentation and production of methanogenesis substrate.

The structure of the rumen microbiome has explained 15–40% of the variation in CH_4_ output in some studies (39, 47), although, it has not yet been determined if the composition of the rumen microbiota influences the methanogenic output of the animal or *vice a versa*. As stated, it is assumed that the presence of individual microbes in the rumen is determined by both ruminal conditions supporting their abundance and the ability of certain microbes to adapt their metabolism to the prevailing conditions in the rumen (48). Some members of the rumen methanogen community have been shown to be heritable (39, 49, 50); however, further work is needed to determine the biological principles of host control over the rumen environment.

The composition and digestibility of the diet offered to ruminants have a major impact on methanogenesis by altering H_2_ concentrations in the rumen, the availability of substrates for fermentation, and the ruminal fermentation profile, which will ultimately influence the availability of substrates for methanogenesis (26, 48). For example, diets high in starch, in comparison to forage, result in elevated production of propionate in the rumen, which results in less CH_4_ being produced per unit of feed intake (10, 26), likely due to the effects of cereals on ruminal pH and passage rate (48). In addition, the digestive physiology of an animal may also influence its methanogenic output, with low MY sheep shown to have a smaller rumen (51).

### The rumen microbiome and methanogenesis

As stated, methanogenesis facilitates ruminal fermentation by reducing the concentration of dissolved H_2_ through the synthesis and eructation of CH_4_ from the rumen (13, 22). Therefore, methanogenesis can be viewed as having a homoeostatic role within the rumen by regulating the concentration of H_2_. However, as the rate of methanogenesis is reduced, prevailing conditions within the rumen favor microbial fermentation pathways, capable of consuming H_2_, at the expense of H_2_-yielding pathways, the net effect of which leads to less H_2_, and ultimately CH_4_, being produced per unit of feed fermented (48). It is also assumed that the presence of individual microbes in the rumen is determined by both ruminal conditions supporting their abundance and/or the ability of certain microbes to adapt their metabolism to the prevailing conditions in the rumen (48). As a result, the composition of the rumen microbial community, including the methanogen cohort, is a reflection of prevailing conditions within the rumen.

The composition rather than the size of the methanogen community in the rumen is closely associated with the methanogenic output of an animal (30). For example, an increased abundance of members of the *Methanosphaera* genera was observed in low-CH_4_-emitting cattle (52–54) and sheep (55, 56). In addition, within the *Methanobrevibacter* genus, Danielsson et al. (52) reported an increased relative abundance of *M. gottcshalkii* and *M. ruminantium* associated with high and low-CH_4_-emitting dairy cows, respectively. Similarly, an increase in the abundance of *M. gottcshalkii* was reported in rumen samples obtained from high MY sheep (56), while an increased abundance of the *M*. RO clade was observed in low RME beef cattle (54).

Martínez-Álvaro et al. (50) proposed that a more diverse methanogen community and an expression of methanogenesis pathways, leading to an increase in competition for methanogenesis substrates among rumen archaea, were associated with a reduced CH_4_ output. In support of this, an increased abundance of methylotrophic methanogens, such as *Methanosphaera*, in low-CH_4_-emitting animals likely is a result of these methanogens having a competitive edge over other groups of methanogens due to their lower H_2_ requirement for methanogenesis (57, 58). Equally, variation within the *Methanobrevibacter* genus, between high and low-CH_4_-emitting animals, has also been considered a product of H_2_ dynamics within the rumen. The *Methanobrevibacter* genus can be segmented into two predominant subgroups, the SGMT (*M. smithii, M. millerae, M. thaueri, and M. gottschalkii*) and the RO clade (*M. ruminantium and M. olleyae*), with the SGMT clade capable of synthesizing both *mcrI* and *mcrII* and the RO subgroup possessing only *mcrI* (30, 59). The expression of both *mcrI* and *mcrII* is regulated by H_2_ availability in the rumen, with *mcrI* and *mcrII* expression occurring in the presence of low and high concentrations of ruminal-dissolved H_2_, respectively (60), which, as depicted by Danielsson et al. (52), likely gives the SGMT clade a competitive advantage in the presence of a greater availability of H_2_.

The presence of individual bacteria in the rumen will be dependent on their ability to alter their metabolism to adapt to the prevailing rumen conditions. Rumen bacteria vary greatly in substrate specificity as well as different groups of bacteria that are associated with H_2_ production and utilization (61). In sheep, Kittelmann et al. (55) identified three different ruminotypes associated with CH_4_ emissions. Ruminotype Q and S were correlated with low-CH_4_-emitting sheep, and they harbored higher abundances of bacterial communities associated with producing propionate and a combination of lactate and succinate, respectively. Ruminotype H contained an increased abundance of bacteria belonging to the *Firmicutes* phylum, including the families *Ruminococcaceae, Clostridiales*, and *Lachnospiraceae*, which contains known producers of CO_2_ and H_2_ (42, 62). In contrast, an increased abundance of lactate-producing bacteria, such as *Sharpea, Kanderila Olsnella*, and *Intestinibaculum* has been reported in low-CH_4_-emitting sheep (55, 63) and beef cattle (54). Coinciding with the relationships of the ruminotypes described by Kittelmann et al. (55), Wallace et al. (64) reported a four-fold increase in the abundance of members of the succinate-producing bacterial family *Succinivibrionaceae* in low- compared to high-CH_4_-emitting beef steers. In addition, high-CH_4_-emitting cattle have been shown to possess a rumen microbiome characterized by an elevated abundance of the butyrate- and formate-producing bacteria *Pseudobutyrivibrio* and *Butyrivibrio* (50, 54).

**Figure 1:**
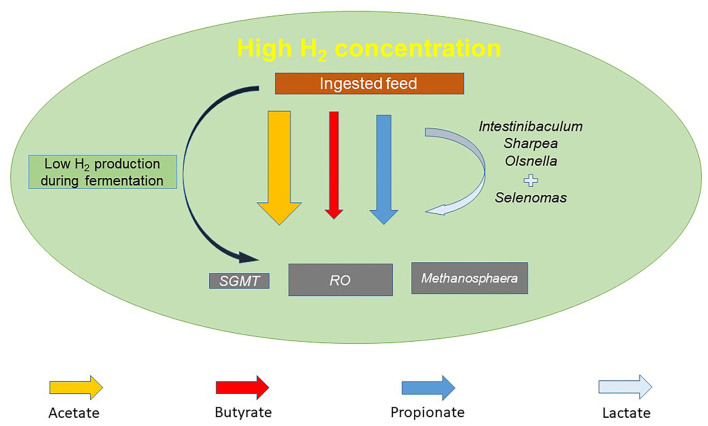
An overview of the rumen microbial community and fermentation profile of a low residual methane emissions (RME) phenotype based on the findings of Smith et al. (54). Differences in the size of the shapes representing individual volatile fatty acids (VFAs) and rumen methanogens are reflective of the dominant VFAs methanogen genera in low RME animals.

Metabolic hydrogen (H) is incorporated into the synthesis of propionate in the rumen with succinate and lactate being the known precursors of the VFA (65). In contrast to this, the stoichiometric calculation of rumen fermentation indicates a net production of metabolic H associated with the synthesis of acetate and butyrate (66) with formate estimated to contribute to 18% of CH_4_ produced in the rumen (67). An increased abundance and/or expression genes involved in the acrylate pathway, including lactate dehydrogenase, lactyl CoA transferase, lactyl CoA dehydrogenase, acyl CoA dehydrogenase, and propionate CoA transferase, has been reported in low MY sheep (63). Greening et al. (68) reported the increased expression of acetyl-CoA synthase and fumarate reductase to be associated with a low-CH_4_ phenotype in sheep. Thus, the presence of individual rumen bacteria and a varied expression of pathways associated with H_2_ utilization and production are associated with the quantity of CH_4_ in animal produce. Figure 1 details the composition of the rumen microbiota of low-CH_4_-emitting cattle based on the findings of Smith et al. (54).

Protozoa have a role in predicting the methanogenic output of an animal as they are key H_2_ producers in the rumen (69). A strong positive linear relationship between log10 protozoal numbers and MY was reported in the meta-analysis of rumen protozoa experiments reported by Guyader et al. (70). Additional meta-analysis studies have estimated defaunation to reduce CH_4_ emissions by 11% but have shown insignificant decreases in methanogen numbers (71). The contrast in reports could be explained by the discovery of the adhesion-like protein (Mru_1499) in *M. ruminantium* M1. The Mru_1499 protein which permits the binding of *M. ruminantium* M1 to the surface of a wide range of rumen protozoa and also to H_2_-producing *Butyrivibrio proteoclasticus* suggests that this methanogen may be capable of continued methanogenesis after defaunation (72). However, the reductions in CH_4_ output associated with rumen defaunation have been reported to be variable, with the practice not being recommended as a mitigation strategy due to its potential negative impact on ruminal feed degradation (9). In addition, the effects of ruminal defaunation on DME have been observed to be short-lived based on data generated from long-term studies (69).

The refaunation work conducted by Belanche et al. (73) showed increased CH_4_ emissions in sheep as they progressed from being protozoa-free to monofaunated and fully faunated. Belanche et al. (73) also showed a greater diversity and 14.4 times greater total abundance of protozoa in their fully faunated animals; however, they did not report statistical differences between the groups in terms of CH_4_ emissions or concentration of methanogens. Furthermore, Kittelmann et al. (55) did not report any distinctive clustering of different ciliate protozoal communities in low- and high-CH_4_-emitting sheep when conducting amplicon sequencing analysis.

Studies in sheep have failed to define clear differences in the clustering of fungal communities of high- and low-emitting animals (55), while studies in dairy cattle have failed to define a definitive relationship between the fungal populations present in rumen samples and CH_4_ emissions (74). In the study by Cunha et al. (74), 73.19% of the fungal samples were identified as unclassified. Therefore, poor taxonomical identification by reference database could be a contributory factor toward the lack of reported correlations between members of the rumen fungal community and methanogenesis. Equally, anaerobic fungi are more commonly found attached to feed particles and thus may be under-represented in studies investigating the microbial community associated with the fluid fraction of the rumen. Work with anaerobic digesters inoculated with fungi originating from the rumen of fistulated cattle showed a positive correlation between fungal numbers and CH_4_ generation (75), perhaps indicating the concentration of the fungal population as a whole to be associated with CH_4_ output.

### Enteric methane measurement technologies for ruminant livestock

The eructation and release of CH_4_ originating from the rumen has led to a variety of techniques being developed to estimate the enteric emissions of livestock. The respiration chamber (RC), the sulfur hexafluoride (SF_6_) tracer method, and the GreenFeed Emissions Monitoring System (GEM) are the most commonly used techniques for estimating enteric emissions in cattle (76). However, as the RC cannot be used to estimate the emissions of animals at pasture, the focus of this review will be on the SF_6_ tracer method and GEM. The RC and additional enteric measurement techniques for ruminant livestock, such as the handheld laser CH_4_ detector and sniffer technique, have been extensively reviewed by others (28). However, as the RC has traditionally been considered the gold standard method for estimating enteric emissions (77), the method, along with the SF_6_ and GEM, is discussed as part of a comparison between the different technologies utilized to estimate enteric CH_4_ emissions from cattle.

### Sulfur hexafluoride tracer technique

The SF_6_ tracer technique for the measurement of enteric CH_4_ emissions was developed by Zimmerman (78), and the use of the method was first published by Johnson et al. (79). An extensive set of guidelines describing the best practice for the use of the SF_6_ method has been published by the Global Research Alliance (GRA) (80). The fundamental concept of this method relies on the biological inertness of SF_6_ gas and its comparable dispersion rate from the rumen to that of CH_4_ (81). Based on such attributes, DME is estimated from the concentration of the mixture of SF_6_ and CH_4_ in an animal's breath.

Permeation tubes of SF_6_ gas, with a known release rate, are administered to the rumen of each animal (82). The release rate of each permeation tube is assessed, prior to administering to each animal, by heating the tubes to 39°C in a water bath and measuring daily mass fluctuation until stabilization (83). After administering the permeation tubes, animals are fitted with a collection apparatus consisting of a harness fitted with pre-evacuated collection cylinders connected *via* a small diameter sample tubing positioned near the nose and mouth of the animal (28, 78). Gases are typically drawn into collection cylinders under partial vacuum (84). Collection tubes are changed normally every 24 h for the determination of gas concentrations using gas chromatography (GC). Samples of the background air are also collected to determine and discount environmental concentrations of CH_4_ and SF_6_ (85).

The below equation is used to determine DME with the differences between the concentration of both gases (SF_6_ and CH_4_) in the collection cylinder, attached to individual animals, and the background cylinders accounted for the following:


$$\begin{array}{l} R_{CH4}=R_{SF6}\frac{{\left[CH_{4}\right]}_{M}-{\left[CH_{4}\right]}_{BG}}{{\left[SF_{6}\right]}_{M}-{\left[SF_{6}\right]}_{BG}}\times \frac{MW_{CH4}}{MW_{SF6}}\times 1000 \end{array}$$

Equation adapted from Williams et al. (86).

R_CH4_ = estimated enteric CH_4_ per animal (g/animal/day); R_SF6_ = previously determined release rate of SF_6_ from the permeation tube (mg/d); CH_4M_ = measured concentration of CH_4_ in collection cylinders fitted to the animal; CH_4BG_ = measured concentration of background CH_4_ in background collection cylinders; SF_6BG_ = measured concentration of background SF_6_ in background collection cylinders; SF_6M_ = measured concentration of SF_6_ in collection cylinders fitted to animal MW_CH4_ = molecular mass of CH_4_; MW_SF6_ = molecular mass of SF_6_; 1000 = conversion factor to account for differential units for SF_6_ (ppt) and CH_4_ (ppm).

### GreenFeed emissions monitoring system

The GEM (C-Lock Inc., Rapid City, USA) created by Patrick Zimmerman (87, 88) is a more recently developed technology for the estimation of enteric emissions from ruminants, with the ability to measure CH_4_, CO_2_, hydrogen sulfide (H_2_S), oxygen (O_2_), and H_2_ from ruminants. It is a non-invasive web-based system that estimates gaseous emissions from individual animals with the use of short-term breath measurements collected throughout the day (89).

**Figure 2:**
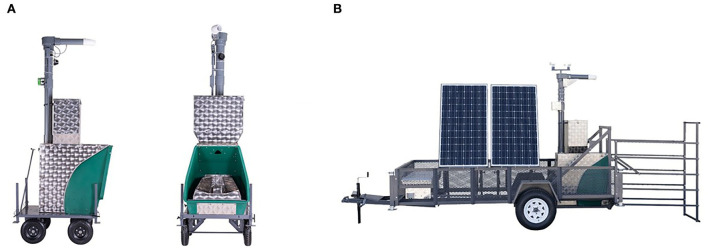
The Greenfeed Emissions Monitoring (GEM) system for **(A)** indoor and **(B)** outdoor use. Image sourced from C-Lock Inc.

A diagram of both the indoor and outdoor pasture-based GEMs is presented in Figure 2. The GEM consists of a head chamber, feed dish, and automatic diet feeder equipped with radio frequency identification (RFID) sensors to identify individual animals fitted with RFID tags (90). When the animal approaches the feed dish, each animal is identified by the GEM *via* their unique RFID tag. A small amount of “bait” feed is dispensed to attract and encourage animals to remain in the system for >3 min (89). As the animal consumes the bait feed, an extractor fan, positioned at the top of the unit, draws a mixture of air that is both exhaled and surrounding the animal's head through the unit and out of an exhaust position at the top of the system (81). Airflow is calculated using a hot film anemometer with a subset of air sampled prior to the outlet (28), with gas concentrations measured using non-dispersive infrared (NDIR) sensors embedded beneath the feed dish (87). Emission measurements are normally taken over a period of 3–7 min, spread across several time points throughout the day, and are dependent on the animal's voluntary visitation to the machine (28), with data uploaded to a web-based interface on an hourly basis.

The concentration of gases emitted by an animal is reported as a gas flux (concentration of an individual gas emitted over a defined period of time; g/day) with the below three equations performed every second (91):


(1) $$\begin{array}{l} Q_{air\left(i\right)}=60\times V_{m\left(i\right)}\times V_{adj\left(i\right)}\times A_{p}\times 1,000 \end{array}$$

Equation 1: Calculation of airflow adapted from Huhtanen et al. (91).

*(i)* = calculation at any time; *Q*_*air*_ = volumetric airflow rate (l/min) calculated on a dry gas basis at 1 unit of atmospheric (atm) pressure; 60 = time conversion; *V*_*m*_ = velocity of air at the center (m/s) of the pipe which is automatically adjusted by sensors to 1 atm pressure; *V*_*adj*_ = is a correction factor (0.88) to account for the *V*_*m*_ to an average pipe velocity; *A*_*p*_ = cross-sectional area of the pipe (0.00811 m^2^); 1,000 = unit conversion.


(2) $$\begin{array}{l} Q_{c\left(i\right)}=\left[C_{p\left(i\right)}\times \left(Conc_{i}-BConc_{i}\right)\times Q_{air\left(i\right)}\right]/10^{6} \end{array}$$

Equation 2: Calculation of gas concentration adapted from Huhtanen et al. (91).

*(i)* = calculation at any time; *Q*_*c*_ = volumetric flow rate of individual gases (l/min); *Cp* = capture rate of air; *Conc* = concentration of captured gas; *BConc* = concentration of background gas; *Q*_*air*_ = volumetric airflow from equation 1; 10^6^ = parts per million (ppm) conversion for gas concentration.


(3) $$\begin{array}{l} Q_{m\left(i\right)}=Q_{c\left(i\right)}\times 273.15\text{/}\left(273.15+T_{air\left(i\right)}\right)\times \rho_{c} \end{array}$$

Equation 3: Calculation of gas flux, based on ideal gas law, adapted from Huhtanen et al. (91).

*(i)* = calculation at any time; *Q*_*c*_ = volumetric flow rate of individual gases (l/min) from equation 2; 273.15 = temperature equivalent in Kelvin; *T*_*air*_ = air temperature (°C); ρ_*c*_ = density of investigative gas at 273.15 K and 1 atm (0.717 and 1.911 g/L for CH_4_ and CO_2_).

### A comparison of methodologies for estimating enteric emissions in a research setting

When operated optimally, all gaseous emissions emitted from the animal (exhaled, eructed, and flatulence) can be quantified with the use of the RC (77). However, the RC is limited to only estimating emissions from a small number of animals within an indoor setting. In addition, animal feeding behavior can be altered by the confined conditions of the RC (28, 77), which may not represent a true reflection of an animal's inherent level of voluntary feed intake and DME.

Both the SF_6_ and GEM method benefit from their ability to be applied in a more natural setting, including at pasture (92, 93), thus having a limited impact on the voluntary feed intake of an animal. Both methods are substantially cheaper when accounting for the greater number of animals that can have their emissions estimated. However, with the SF_6_ technique, animals require daily handling for the replacement of collection cylinders, which may alter feeding behavior (94). The SF_6_ method is labor-intensive and very much dependent on the availability of local expertise to manage the equipment (82). In addition, the permeation rate of SF_6_ from boluses administered to animals can vary (28); however, modifications to the technique, such as the addition of a flow restrictor as proposed by Deighton et al. (84), have improved accuracy. The SF_6_ method is also noted as being a less robust technique with frequent equipment failure (95).

In contrast to the SF_6_ tracer method, the GEM is more user-friendly and has the ability to test a greater number of animals simultaneously. However, the GEM is reliant on individual animals voluntarily visiting the unit. Therefore, animals will require an acclimatization period to ensure habituation and regular attendance at the GEM (93). In addition to this, the effects of the bait feed, used to encourage animals to use the GEM, on CH_4_ output must also be accounted for, particularly when investigations are conducted at pasture (28). Furthermore, emission data are not continually captured from each animal with the method reliant on the collection of measurements over several days. It is reported within the literature that animals will be required to visit the GEM 30–50 times for >3 min or have the emissions evaluated over 2–5 weeks (89, 96–98) for an accurate estimation of DME. With the use of the GEM, it is imperative that the methodology implemented by researchers promotes an even visitation to the unit over a 24h period to reflect the diurnal pattern of enteric emissions (77). Recently, some authors have advocated for the use of daily CO_2_ emissions (DCE; kg/day) as a proxy for DMI due to the linear relationship observed among both traits (99). As DCE can be measured with the use of the GEM, this technology may have a subsequent benefit in allowing for an approximate estimation of the voluntary feed intake of ruminants at pasture.

Between animal variation for CH_4_ output (both DME and MY) tends to be lowest with the use of the RC (94, 100); however, some studies have shown the opposite for the GEM (76, 101). A lower DME when RCs are utilized as the reference methodology for quantifying enteric emissions in comparison with both the GEM and SF_6_ method has been reported in some studies (94, 100, 101), which may be reflective of the impact of the RC on animal feeding behavior. For example, in the meta-analysis of forage-fed cattle conducted by Jonker et al. (94), the mean DMI and DME for cattle measured in the RC (8.2 kg/day; 187 g/day) were observed to be substantially lower in comparison to when it was quantified with the GEM and SF_6_ method (12.1–13.3 kg/day; 235–292 g/day). However, when a degree of feed restriction is applied during the estimation of emissions with all techniques, an increased DME was observed with the use of the RC in comparison with the GEM and SF_6_ method (76), which in part, is likely reflective of CH_4_ released *via* flatulence being accounted for with the RC.

Velazco et al. (102) reported no statistical difference in CH_4_ output estimated by the RC or GEM, with a strong and moderate correlation between methods for DME (*r* = 0.85) and MY (*r* = 0.58). Similarly, no difference in MY and comparable CV % was detected when measurements were performed on dairy cows using the modified SF_6_ and RC (84). Estimates of MY between the GEM and RC did not differ for low RFI animals in Alemu et al. (101). A weak and non-significant correlation between the RC and GEM for estimates of MY (*r* = 0.32) was detected by Doreau et al. (76) however, a strong relationship was determined for the SF_6_ and GEM (*r* = 0.72). Garnsworthy et al. (100) reported a correlation of (*r* = 0.87) and (*r* = 0.81) for SF_6_ and GEM estimates of DME with the RC, respectively.

Poor agreement in estimates of MY between the SF_6_ and GEM has been reported (76, 94) while DME was observed to be moderately correlated (*r* = 0.40) (100). Differences in estimates of enteric emissions between both methods, could be due to CH_4_ which is absorbed in the bloodstream and exhaled with it currently unknown if SF_6_ and CH_4_ diffuse across the rumen wall at the same rate (76). The CV% for CH_4_ output has been reported to be greater with the SF_6_ method in comparison with the GEM (76, 90, 100). However, the GEM will require an increased measurement period and sample size, in comparison with the SF_6_, to ensure animals attend the unit an appropriate number of times to ensure accurate data collection (28).

Both the SF_6_ and GEM can accurately estimate enteric emissions from ruminant livestock, with the ultimate choice of methodology reflective of the type of research being conducted (28). However, regardless of the method deployed, rigorous calibrations are warranted to ensure accurate data are collected (28).

## Methane mitigation strategies for pastoral-based beef cattle production

Reducing age at slaughter, and thus decreasing the lifetime emissions of an animal, has been advocated as one of the most effective CH_4_ reduction strategies for beef cattle production (103, 104). A reduction in the lifetime emissions of the animal can be achieved *via* the implementation of practices that directly hinder the methanogenesis process and thus reduce the quantity of CH_4_ an animal emits on a daily basis. Strategies that reduce daily CH_4_ emissions will be of dual benefit in a calf–cow production system as they can likely be utilized to reduce the quantity of CH_4_ emitted from both the cow and calf. Alternatively, strategies that increase the daily weight gain of growing animals have the potential to reduce the time it takes for cattle to reach slaughter weight, which in turn will decrease the overall quantity of CH_4_ an animal emits over their lifetime (103). Indeed, in the latter scenario, a slight elevation in DME, associated with the necessary increase in DMI likely required to facilitate a superior growth rate, can be tolerated as long as a reduction in lifetime emissions is achieved.

Both alterations to the diet offered to beef cattle and genetic selection have the potential to reduce the lifetime emissions of beef cattle. Improving the quality of the diet offered to ruminant livestock will likely increase ADG, but depending on the strategy employed, it may also lead to a direct reduction in DME. Equally, through animal genetics, it may be possible to select animals with an inherently lower methanogenic output or enhanced growth rate. In addition, the simultaneous selection for improved animal growth rates and reduced CH_4_ output may be feasible with the use of a multitrait genetic selection breeding program. Furthermore, an anti-methanogenic vaccine would be mutually beneficial for both breeding and finishing animals. Below, a review of the most effective and practical CH_4_ abatement strategies for pasture-based beef cattle production within temperate climatic regions is presented.

## Dietary-based methane mitigation strategies

The delivery of even some of the most potent antimethanogenic additives to pasture-based livestock will be a challenge for the sector. Some additives may be included as part of a supplementary ration offered during the finishing period, included as part of a winter feeding regime or supplemented to the lactating beef cow. Nonetheless, with forage accounting for 90% of the diet of some pasture-based beef cattle production systems (105), management of the grazing sward, to either reduce enteric CH_4_ emissions or boost animal growth rates, will likely be the most practical means of reducing the lifetime emissions of beef cattle. A summary of the proposed mode of action for dietary-based CH_4_ abatement strategies, discussed in this review, is presented in Table 2.

**Table 2:** Description of the antimethanogenic mode of action of dietary-focused methane abatement strategies.

**Strategy**	**Mode of action**	**Limitations**
Grain and cereals	Promotes propionate production and lowers ruminal pH	Total CH_4_ does not always decrease. Cereals could be used as source of human food
Improved forage quality	Potentially due to increased digestibility of forage	Knowledge of impact on rumen microbiome limited. May increase daily CH_4_ emissions due to increased feed intake
3-NOP	Limits methanogenesis by binding to the active site of the *mcr*	Difficulties in supplementing animals at pasture and lack of details on cost
Forbs and herbs	Unknown. Potentially due to increased forage digestibility and condensed tannins in some forages	May increase daily CH_4_ emissions due to increased feed intake and issues with persistency in the grazing sward
Lipids	Negative impact on cellulolytic bacteria and the rumen methanogen community	Potentially a costly strategy and limited to a max inclusion rate of 6–7% in the diet to prevent issues with the fermentation of ingested forage
Legumes	Unknown. Potentially due to increased forage digestibility or condensed tannins in some legumes	May increase daily CH_4_ emissions due to increased feed intake and issues with persistency in the grazing sward as well as bloat

### Management of the grazing sward

In the rumen, the fermentation of the fiber is a complex process, involving a combination of fibrolytic microbes (5). An increase in the concentration of fiber within grazed forage can negatively influence the ruminal fermentation of ingested plant matter due to its slow and sometimes indigestible nature (106). In addition, a reduction in sward digestibility has the potential to increase gut fill, which will likely result in a reduced voluntary feed intake (107) and, potentially, animal productivity. Equally, an increased fermentation of fiber promotes a greater acetate:propionate (A:P) ratio within the rumen, which is known to facilitate a greater rate of methanogenesis per unit of feed intake (108).

Low pre-grazing herbage mass (HM) swards (due to the plant being in a stage of regrowth) have been shown to have lower structural fiber content and greater digestibility (109, 110). As a result of their increased digestibility, low HM swards have been shown to facilitate greater consumption of grazed forage and concurrent positive responses to animal growth rates in some studies. For example, Boland et al. (111) observed an average increase in ADG of 17% when the herbage mass of the grazing sward, offered to Limousin X heifers, reduced from 2,000 to 1,300 kg of DM and 3,200 to 2,800 kg of DM. Although HM had no impact on DME, the environmental efficiency of animal growth improved at lower herbage masses, with a 17 and 22.6% reduction in MADG observed in 1,300 and 2,800 kg of DM swards, respectively. In addition, increased production of VFA was observed on the low HM swards. In contrast to this, however, Doyle et al. (112) recently observed a reduced ADG due to lower consumption of feed, when yearling steers grazed swards with an HM of 1,500 kg of DM in comparison to 2,000 kg of DM. As a result, further analysis of the effects of pre-grazing HM on the lifetime emission profiles of grazing beef cattle is warranted. Equally, the effect of HM on the production of animal protein per unit of land area must also be considered. At a lower HM, the reduced availability of forage will decrease the number of animals that can be produced per unit of land area. However, decreasing the time it takes to slaughter animals can free up land, which can subsequently be utilized to graze additional animals, leading to an increased overall production of animal protein per unit of land and potential improvement in farm profitability.

The nutritional quality of grazed forages can differ between extensive and intensive grassland production systems. Both Fraser et al. (113) and Richmond et al. (114) observed an increased structural carbohydrate content of uphill swards in comparison with well-managed lowland swards. As expected, due to differences in the nutritional quality of both sward types, animals grazing the lowland swards had higher growth rates, which were likely supported by greater consumption of forage. While daily DME was increased in lowland swards due to an elevated feed intake, greater ADGs reduced MADG in both studies by 25–38%.

Grazing platforms with higher stocking rates (i.e., number of animals/livestock unit per ha) have been shown to produce higher quality forage due to pastures being grazed at a lower sward height (115). Equally, rotational grazing is often utilized as a management practice to target grazed grass when it is in a stage of regrowth. In spite of this, there are conflicting reports of the effect of both stocking rate and grazing management on the quantity of CH_4_ emitted by individual livestock. Pinares-Patiño et al. (116) reported no overall difference in DME or MADG when stocking rates increased from 1.1 to 2.2 LU/ha. Similarly, Chiavegato et al. (117) reported no difference in enteric CH_4_ output of lactating Angus cows between stocking rates of 1 and 2.5 cows/ha. However, rotational grazing systems have been shown to lower the DME of beef heifers and support higher growth rates (118). Conflicting reports have been observed in sheep, with animals grazing within a continuously stocked system shown to have an increased ADG and reduced CH_4_ intensity (119), albeit similar results have not been replicated in grazing beef cattle. As a result, for beef cattle, it would appear that grazing management is likely to have a greater influence on the enteric CH_4_ output of individual animals in comparison with the stocking rate, although more research is required in this area.

### Methane mitigation potential of different varieties of perennial ryegrass

Perennial ryegrass (PRG) is one the most common species of grass utilized in pasture-based livestock production due to its superior yield and persistence with the grazing platform (5). With selective breeding, PRG cultivars with an increased water-soluble carbohydrate (WSC) content are now currently available (120). Water-soluble carbohydrates are rapidly fermented in the rumen and have been shown to promote a greater production of propionate at the expense of acetate and lower ruminal pH (121, 122). As a consequence of the aforementioned alterations to the ruminal fermentation profile, increasing the WSC content of a forage-based diet has been shown to reduce enteric CH_4_ emissions per unit of dry matter digestibility (DMD) by up to 14% *in vitro* (123). Furthermore, *in vivo*, offering forages with an increased WSC:NDF to ruminant livestock has been associated with reduced methanogenic output and is shown to account for some 38% of the variation in MY across studies (124). Although data are scarce in beef cattle, elevated growth rates have been reported in sheep (121, 125) offered forages with an elevated WSC content. Reports on the CH_4_ output of animals grazing swards with an increased WSC have been mixed, with some (125) but not all (126, 127) studies noting a reduction. Based on the literature, reductions in CH_4_ output have only been observed when the WSC:NDF of PRG varieties was substantially elevated. Nonetheless, if the positive effect of high WSC PRG on the growth rate of lambs can be translated to beef cattle, high-sugar grasses may have the potential to reduce days to slaughter and the lifetime emissions of beef cattle.

As discussed in subsequent sections, increasing the lipid content of the diet offered to ruminants is one of the most established CH_4_ abatement strategies for ruminant livestock (9, 128). Researchers in New Zealand have made strides to increase the fatty acid content of PRG *via* gene modification (129). Promising *in vitro* data suggest that high lipid grass can reduce enteric CH_4_ production by ~15% without impacting total VFA production (130). Recent data also suggest minimal differences in the establishment between high and low PRG swards (131). Pending results from *in vivo* trails, the utilization of high-lipid grasses would be an attractive CH_4_ mitigation solution for grass-based livestock production systems.

### Incorporating clover into the grazing sward

Both white (*Trifolium repens*) and red *(T. pratense)* clovers are often included in the grazing sward due to their unique ability to fix atmospheric nitrogen (N) into usable means for plants and thereby increase herbage production (132–134). As a result, the incorporation of white clover into the grazing sward is considered a cost-neutral nitrous oxide (N_2_O) mitigation strategy for temperate grassland production systems (103).

The fermentation of both red and white clover has been associated with a reduced level of CH_4_ production in comparison with PRG and other grass species *in vitro* (135, 136). Furthermore, a reduction in the total number of ruminal methanogens (*in vitro*) and a reduced relative abundance of *Methanobrevibacter* (*in vivo*) have been observed with red and white clover, respectively (136, 137). However, data on the antimethanogenic benefits of clover *in vivo* have to date been limited, with studies conducted in beef cattle lacking. In both grazing (dairy cows) and indoor feeding trials (sheep and dairy cows) investigating the antimethanogenic effects of red and/or white clover, elevated DME has been reported in some (138–140) but not all studies (141). The heightened digestibility of white clover likely results in its rapid passage through the rumen (142), facilitating a greater DMI in comparison to grasses (138, 140, 142). As DME is strongly influenced by feed intake (45, 46, 143), the observed increase in DME in some studies, associated with ruminants grazing white clover/PRG (WCPRG) swards or offered increasing amounts of clover, is likely due to the legumes' ability to support higher feed intakes. To date, reported improvements in the growth rate and milk production have been reported in grazing sheep and dairy cattle (144–147), but not beef cattle (148).

It is evidentially clear from the literature that there is a dearth of published data on the effects of clover on both the performance and methanogenic output of beef cattle. Nonetheless, due to N_2_O mitigation benefits associated with the legume, clover is likely to contribute to a reduction in total on-farm emissions. Furthermore, work is currently ongoing to increase the tannin content of white clover, which may not only help alleviate the incidence of bloat in grazing ruminants but also reduce enteric CH_4_ emissions. Tannins are secondary plant compounds that were traditionally investigated due to their ability to reduce rumen degradable protein (149, 150), with condensed tannins (CTs) also shown to have a negative impact on rumen methanogens and reduce the degradation of plant polymers (151, 152). In comparison with other legumes, the CT content of clover plants is low, with the majority of the compound concentrated in the flower of the plant (153). However, transgenic research is currently underway to increase the availability of CTs in the leaf of white clover, which has been shown to produce 19% less CH_4_ than traditional varieties *in vitro* (154). Assuming similar results are observed *in vivo*, high CT white clover could be a potential mitigation solution for pastoral-based production systems, albeit the impact of modified clovers on animal productivity will require assessment.

### Alternative legumes and herbs

The combination of herbs and alternative legumes as part of a mixed species grazing platform has been shown to increase N-use efficiency in dairy cows (155), reduce the burden of intestinal parasites in lambs (156), and promote greater milk yields (157) and animal growth rates (156, 158). In addition, mixed species swards have also been shown to have a lower N requirement to PRG swards (147) and reduced N_2_O emissions (159). As a result of the benefits to animal performance and health, as well as sward digestibility and reduced N_2_O emissions, interest has increased in the number of studies investigating the antimethanogenic potential of mixed species swards.

Dairy cows grazing swards, containing predominantly the herbs chicory (*Cichorium intybus*) and plantain (*Plantago*), have been reported to tentatively emit 15% less CH_4_ in comparison with cows grazing WCPRG pastures (155). Similarly, offering zero-grazed chicory, in comparison with WCPRG, resulted in a 37% reduction in MY, but a comparable level of DMI, in sheep (160). However, contrasting results have been reported when both herbs have been offered to sheep as part of other trials, with no effect on DME reported in some studies (161, 162). Williams et al. (163) reported increased MY and reduced milk production in dairy cows offered lucerne (*Medicago sativa*) and chicory in comparison with cows receiving lucerne only. However, reproductive stage chicory was offered in the previous study, which could suggest that similar to other forage species, the methanogenic output of animals offered chicory may fluctuate throughout the grazing season. Any potential antimethanogneic effects of both plantain and chicory are yet to be defined as neither has been reported to have a high CT content, but could potentially arise from a lower fiber content within herbs (164). Importantly, reports of elevated production of CH_4_ have been limited in the data produced to date when both plantain and chicory are simultaneously combined as part of a mixed species sward. However, similar to investigations of the CH_4_ abatement potential to white and red clover, there is a dearth of information on the effects of both herbs on the CH_4_ output of beef cattle.

Both lucerne and birdsfoot trefoil (BT; *Lotus corniculatus*) are alternative legumes that have been investigated in temperate regions. Lucerne, has to date, shown little promise as forage capable of reducing DME, with similar emissions to that of grasses reported in heifers (165) and goats (166). Nevertheless, in the extensive review published by Popp et al. (167), lucerne was shown to improve the weight gain of cattle across numerous studies. Although a greater number of studies investigating the effects of lucerne on CH_4_ in beef cattle are warranted, the reported improvements to the growth rate of beef cattle suggest that the legume may have a role in reducing the lifetime emissions of an animal by improving the environmental efficiency of animal growth. In comparison, BT is noted as having a high proportion of CTs (168). Increased milk yield and reductions to MY have been observed when BT silage was offered to dairy cows in comparison with PRG silage (169). In addition, sheep offered BT produced 33% less CH_4_ compared with those offered PRG, with a minimal difference in DMI (169). *In vitro*, a simultaneous reduction in CH_4_ output and decreased abundance *Methanobrevibacter* was observed for BT in comparison to Orchardgrass (170). However, in the latter study, a reduction in the abundance of cellulolytic bacteria *Fibrobacter succinogenes* and *Ruminococcus flavefaciens* was associated with BT. The negative impact of BT on these key cellulolytic bacteria, in particular *F. succinogenes*, likely originates from a limited degradation of plant polymers as a result of CTs forming indigestible complexes with plant polymers such as cellulose, hemicellulose, and pectin in the rumen (149).

### Forage brassicas

Brassicas, primarily consisting of kale (*Brassica oleracea* L. cv. Kestrel), turnip (*B. campestris* L. cv. Appin), rape (*B. napus* L. cv. Titan), and swede (*B. napus* L. cv. Dominion) are annual forage crops that have traditionally been utilized in temperate geographical regions as feed sources for out-wintered livestock or during periods of herbage deficit due to summer drought (171). In comparison to grasses, forage brassicas are noted as having an increased digestibility, WSC content, and lower proportion of fiber (171, 172). As a result, the ruminal fermentation of some brassica crops has been shown to boost animal productivity (171) and illicit an alteration in the rumen fermentation profile associated with elevated production of propionate and subsequent reduction of ruminal pH (173). Sun et al. (172) assessed the CH_4_ mitigation potential of different brassicas against PRG in sheep. In their analysis, both forage rape and swedes were shown to have the lowest MY; however, forage rape had the least impact on DMI. As part of numerous additional studies, across all ruminant species, forage rape has been shown to promote a lower MY (174). For example, up to a 40% reduction in MY was observed in beef heifers offered winter forage rape in comparison with pasture (175). In addition in sheep, a linear reduction in both DME and MY was observed as increasing proportions of forage rape replaced PRG (0, 25, 50, 75, and 100%) in the diet. Indeed, when forage rape was offered as the sole forage in the previous study, DME and MY were reduced by 55 and 64%, respectively. The antimethanogenic effects of forage rape have also been shown to persist for over 3 months, with lambs consuming forage rape as the sole component of their diet, on average emitting 22–30% lesser CH_4_ per unit of feed intake in comparison with lambs offered PRG after 15 weeks (176).

Forage rape likely reduces ruminal methanogenesis due to the rapid fermentation of the crop, having a negative effect on the rumen methanogen community as a result of elevated production of propionate and lower rumen pH (176). The rumen microbial community of lambs consuming forage rape has been shown to have a greater abundance of the rumen bacteria *Selenomonas* and *Sharpea* and an increased abundance of the methanogen *Methanosphaera* (176), similar to the microbial profiles previously associated with low-CH_4_-emitting beef cattle (54) and sheep (56). In addition, a reduced rumen protozoal abundance was also observed in sheep offered forage rape (176). Some authors have also argued that secondary plant compounds in forage rape, such as glucosinolates, may reduce methanogenesis by increasing the ruminal passage rate (177). As reported by Sun (176), forage rape has also been shown to have a nitrate concentration that is 10 times greater than PRG (176), with the ingestion of nitrate in the rumen known to have a negative impact on ruminal methanogenesis (178, 179). However, nitrate is not believed to contribute to much of the antimethanogenic effects of forage rape (176). The annual persistence of forage rape will likely be its major limitation of the crop being utilized as an antimethanogenic forage source. However, the persistent reductions in daily CH_4_ production and beneficial responses to animal growth rates observed across numerous studies may see an increased interest in forage rape as an antimethanogenic strategy for some pasture-based beef producers.

#### Methane mitigation potential of dietary additives for pasture-based beef cattle

Numerous CH_4_ abatement dietary additives have been identified to date (128). However, supplementing pasture-based beef cattle with antimethanogenic compounds will be a challenge. High energy substrates are routinely supplemented to the diet to increase muscle growth during the finishing period, meet the energy demands of pregnancy/lactation, and overcome deficiencies in nutrient supply in some production systems (180). Therefore, the addition of lipids and other antimethanogenic compounds could be administered at different time points throughout the production cycle *via* a supplementary concentrate feeding regime. Although it is acknowledged that strategies directly focused on pasture will have the greatest mitigation effect within a grass-based beef system, some mitigation benefits at different stages of the animal's life may arise from the supplementation of concentrates formulate with antimethanogenic compounds. Equally, all dietary supplements can theoretically be administered to ruminant livestock during a winter housing period.

##### Grain supplementation to forage based diets

An increase in the digestibility of dietary constituents is known to decrease the quantity of CH_4_ emitted per unit of feed intake (181). This can likely be explained by the model of H_2_ dynamics proposed by Janssen (48), whereby a reduction of rumen pH and increased passage rate associated with concentrate supplementation is likely to have a negative impact on the rumen methanogen community, ultimately leading to a reduction in methanogenesis. In addition, the feeding of cereals elevates dissolved H_2_ concentrations in the rumen, which results in a redirection of metabolic H to the synthesis of propionate (48).

As alluded to in previous sections, an increase in the digestibility of the diet offered to ruminants can facilitate an enhanced level of feed intake, with increases in DMI observed in numerous studies when concentrates are offered in the place of forage (42, 182, 183). As a result, some studies have shown that when concentrates are supplemented, DME is either increased or is comparable with high-forage diets (183–185). In contrast, other studies have noted reductions in DME when the forage proportion of the diet is substituted with concentrates (42, 182, 186). However, in the latter studies, with the exception of Rooke et al. (42), although only ~1 kg difference was observed, increasing the proportion of forage in the diet had very little impact on feed intake. Therefore, under scenarios whereby a substantial increase in DMI is associated with concentrate supplementation, a subsequent elevation in DME is likely to occur. However, when supplementation has no effect on DMI, a reduction in DME is expected, with a lower MY generally observed when concentrates account for 35–40% of the diet fed to ruminants (9, 187).

Recent life cycle analysis (LCA) has shown that although MI may be less for finishing systems where concentrates are supplemented at various levels, overall emissions are lower for grass-only finishing systems due to a reduction in emissions arising from the production of animal feed (188, 189). However, in the previous study, all animals were slaughtered at a common age (20 months) with concentrate-supplemented animals producing a heavier carcass in comparison with grass-only animals. Therefore, within a system whereby animals are slaughtered based on a targeted finishing weight, supplementing concentrates to pasture-based animals can reduce the age of slaughter, albeit the impact to on farm profitability will need to be considered.

##### Increasing the lipid content of the supplemented ration

Traditionally fat supplementation has been utilized as a means of increasing the energy content of ruminant diets. However, supplementing the ruminant diet with fat has been shown to reduce CH_4_ output in numerous studies (190–194), with meta-analysis conducted by Patra (195) and Beauchemin et al. (196) indicating that for every 1% increase in the lipid content of the diet, DME and MY are predicted to decrease by 3.77 and 5.6%, respectively. The supplementation of lipids, in particular to cattle consuming a high forage diet, has been shown to have a negative impact on fiber digestion and feed intake in beef cattle (190, 191). As a result, fat supplementation is generally recommended not to exceed 6–7% (197) in an effort to negate fiber fermentation and palatability issues.

Medium-chain fatty acids (MCFAs) and polyunsaturated fatty acids (PUFAs) have been observed to have the most potent antimethanogenic capacity (11). However, due to the cost associated with the processing of MCFAs, the use of PUFAs may be a more economical option (196). The supplementation of unsaturated fatty acids was traditionally believed to reduce ruminal available H_2_ for methanogenesis *via* biohydrogenation (26). However, the biohydrogenation of unsaturated fatty acid is likely to only to absorb 1–2% of H_2_ production (9). The antimethanogenic effect of fats is believed to originate from the negative effect of lipids on cellulolytic bacteria leading to a reduction in organic matter digestibility (OMD) and a negative impact on the rumen methanogen community *via* a diversion of H_2_ away from methanogenesis (11, 191). Furthermore, rumen microbes possess a limited ability to ferment lipids (198). As a result, the addition of lipids to the diet, at the expense of other carbohydrate sources, likely results in a lower availability of fermentable substrate, leading to less VFA, H_2_, and ultimately CH_4_ production per unit of intake and/or digestibility (199). The supplementation of lipids has been identified as a costly CH_4_ mitigation strategy (103), which may limit its use due to its economic viability (10). However, the processing of crops for the global food, oil, and ethanol industries generates a source of residual byproduct (BP) plant matter, which can be utilized as cheap animal feed (200). Indeed, the nutritional value between BPs is known to vary, with some shown to have a high oil content (201). For example, dried distillers grains plus solubles (DDGS), originating from maize, have been reported to have a fat content of 9–12.7% (202–204). Replacing barley with DDGS, as part of the diet offered to beef cattle, has been shown to reduce enteric CH_4_ output by >19% by McGinn et al. (203). Increased demand for biodiesel has promoted a more efficient oil extraction process from maize, with the lipid content of refined DDGS reported to be 30% less than traditional forms of DDGS (205). Nonetheless, evidence from the literature still highlights the CH_4_ abatement potential of refined DDGS, albeit it may be slightly reduced in comparison to unrefined forms. For example, the formulation of concentrates with either DDGS or refined DDGS has been shown to support similar reductions in MY in dairy cows (206). In addition, supplementing concentrates formulated from refined DDGS to dairy cows supported a 6.4% reduction in DME with no impact on DMI or milk yield (207). Furthermore, supplementing rapeseed cake, in place of rapeseed meal, reduced MY by 7% in dairy cows (208). Therefore, increasing the fat content of the diet offered to ruminants may be economically achieved by the supplementation of BPs.

However, the high protein content of some plant-based BPs, such as DDGS, has increased N excretion in some studies (209, 210), which may offset any reductions in enteric emissions and lead to a net increase in farm gate GHGs (211). Subsequently, this issue may be overcome by formulating rations from a combination of BPs, with both Whelan et al. (212) and Condren et al. (213) reporting no differences in N excretion between dairy cows receiving a barely and soybean meal supplemented ration in comparison with a BP ration formulated from DDGS, palm kernel expeller, and soyhulls. Indeed, the same BP formulation was also shown to reduce CH_4_ output by ~20% (both daily and per unit of OMD) without negatively impacting members of the rumen microbiota when supplemented with a pasture-based diet *in vitro* (199).

##### Supplementation of 3-nitrooxypropanol to ruminants

The synthetic compound 3-nitrooxypropanol (3-NOP; DSM Nutritional Products Ltd., Kaiseraugst, Switzerland), Bovaer^®^ as it is commercially known, produced by DSM, has been demonstrated to be a non-toxic antimethanogenic supplement across numerous studies in dairy and beef cattle (214–220). 3-Nitrooxypropanol binds to the active site of the *mcr* enzyme, rendering it incapable of completing the final methanogenesis step (221). Unsurprisingly, this supplement has been shown to negatively affect members of the methanogen population. At a 2.5-g of 3-NOP/animal/day supplementation rate, 5.6- and 5.5-fold reductions in the total methanogen population and *Methanobrevibacter* spp. were accompanied by a 30% in DME in the study conducted by Martinez-Fernandez et al. (216). Similarly, Romero-Perez et al. (215) observed a reduction in the total methanogen population when 3-NOP was supplemented daily at 2 g/animal.

Reductions in CH_4_ production as high as 59% have been reported in some studies when 3-NOP was supplemented in cattle (215). Increasing the supplementation rate of 3-NOP linearly decreases CH_4_ emissions (222) with an average reduction in DME and MY of 32.5 and 29.3%, respectively, reported in the meta-analysis conducted by Dijkstra et al. (223) based on a mean supplementation rate of 123 mg of 3-NOP/kg of DMI. The meta-analysis by Dijkstra et al. (223) also suggested that 3-NOP is more potent in dairy compared with beef cattle, with on average, a lower dosage (per unit of intake) capable of achieving greater reductions in CH_4_ output in dairy animals. However, as dairy cows are known to have a higher intake in comparison to beef animals, it may be more appropriate to compare animals based on the total dosage of 3-NOP per day rather than per unit of intake.

Overall, there have been no reports of a negative effect of 3-NOP supplementation on animal productivity in studies conducted on beef cattle. In the recent meta-analysis of some 14 studies, Kim et al. (224) noted a slight reduction in DMI in beef cattle supplemented with 3-NOP. In addition, in the early meta-analysis conducted by Jayanegara et al. (222), a small increase in gain to feed (G:F) was reported in animals receiving 3-NOP. Similarly, in their feedlot study consisting of over 4,000 beef cattle, Alemu et al. (220) reported that animals receiving a 3-NOP dosage of 200 mg/kg DM had a 2.6% reduction and a 2.5% increase in DMI and G:F, respectively. The lack of a substantial benefit to animal productivity with the reduction in enteric CH_4_-associated 3-NOP's supplementation is somewhat unexpected, but could partially be a result of the fact that 3-NOP does not increase the energy concentration of the diet unlike other anti-methanogenic additives, such as lipids. However, considering methanogenesis is estimated to consume 2–12% of the gross energy content of the diet consumed by ruminants (26), a positive response to ruminant performance would be anticipated as CH_4_ production decreases. Some authors have noted the slight reductions in DMI associated with 3-NOP could be due to the reported increase in ruminal propionate production (225). Indeed, an increase in the concentration of ruminal propionate production can have a hypophagic effect on ruminants and thus impact both satiety and hunger (226, 227). Therefore, while propionate is a more energy-dense ruminal VFA in comparison to acetate (C2 vs. C3), the lack of a substantial benefit to animal performance may be explained by the negative effect 3-NOP has on DMI and the negligent effect on GEI, which likely counteracts any potential major gains to animal productivity associated with a reduction in energy lost to methanogenesis. However, in the meta-analysis of Kim et al. (224), 3-NOP supplementation only had a negative effect on DMI, but increased propionate, in beef cattle studies. However, as eluded to by Yu et al. (225), the effect on DMI may be due to the higher 3-NOP inclusion rates utilized in beef in comparison to dairy studies, which could indicate issues with palatability at higher dosage rates. Nonetheless, this does not explain the discrepancy in the varied effects of 3-NOP on ruminal propionate and the associated impact on animal performance, as when 3-NOP was supplemented to target an 80% reduction in DME *in vitro*, Guyader et al. (228) observed no impact on ruminal propionate production. Subsequently, in Guyader et al. (228), an increase in other reduced fatty acids, such as valerate and caproate, along with elevated gaseous H production was observed with 3-NOPs supplementation. Coinciding with this, an increased redirection of ruminal H toward valerate and caproate production, but not propionate was observed. Consequently, when all H production and utilization pathways were assessed, only 54.3% of ruminal H was accounted for, indicating that the excess H associated with 3-NOPs supplementation was not redirected to the main ruminal fermentation pathways and was ultimately expelled as gaseous H (228). Therefore, further work is required to better understand the effects of 3-NOP's supplementation on the main rumen microbial fermentation pathways and the effects this has on ruminal H.

Van Wesemael et al. (219) confirmed the ability of 3-NOP to have a similar mitigation effect when mixed into the forage diet or supplemented in a formulated concentrate. However, the antimethanogenic effects of 3-NOP are short-lived, with enteric emissions shown to increase once supplementation has ceased (214) and the compound is no longer available within the rumen. As a result, even if it was included as part of a supplementary ration, in its current form 3-NOP will likely have minimal impact on reducing the methanogenic output of grazed beef cattle. Slow-releasing forms of 3-NOP are currently under investigation for the use of the compound within a pastoral setting (229), which may deliver some benefit in the future. Currently, Brazil, Chile, and the EU (dairy cows only) are the only countries that have granted regulatory approval for the use of 3-NOP on farms. While 3-NOP is no doubt a very promising CH_4_ mitigating compound, further investigation is warranted to assess the economic availability of its inclusion in ruminant diets as well as the slow-release versions of the compound that can be utilized within a grass-based system.

#### Anti-methanogenic vaccinations

The development and utilization of anti-methanogen vaccines, as a CH_4_ mitigation strategy, is an attractive prospect for the livestock industry. Indeed, the inoculation of ruminants with an antimethanogen vaccine is likely to be among the most popular mitigation strategies among livestock producers, with a high uptake at the farm level, if proven effective (230). The mode of action of an antimethanogenic vaccine is likely to be through the synthesis of antibodies, which enter the rumen *via* saliva and subsequently target a methanogen cellular surface antigen which inhibits the methanogenesis process (231). Antibody responses following the administration of anti-methanogenic vaccinations have been reported (232); however this has not always led to a reduction in CH_4_ output (233, 234). Wright et al. (235) observed a 7.7% reduction in MY after sheep were administered a vaccine formulated to target three strains of the *Methanobrevibacter* genus. With advances in NGS and bioinformatics, reverse vaccinology, whereby potential target antigens are detected by studying the genomes of rumen methanogens (231) may benefit the development of antimethanogen vaccines in the future. However, efforts thus far to develop an effective antimethanogenic vaccine have been limited, primarily due to difficulties in the large diversity of the methanogen community residing in the rumen (236).

#### Animal breeding strategies for methane mitigation

As previously discussed, CH_4_ output is a heritable trait presenting the opportunity to breed low-CH_4_-emitting animals. However, CH_4_ output is a complex trait under the influence of multiple factors and is both phenotypically and genetically correlated with DMI, ADG, and body weight (37, 38, 44). Therefore, directly selecting animals for a reduced DME phenotype will likely decrease animal performance and therefore be unfavorable among producers due to negative ramifications to on-farm profitability.

The selection of low-CH_4_-emitting cattle on the basis of ratio traits, such as MY, has traditionally been advocated as MY was perceived to be free from any association with feed intake or body weight but positively correlated with DME, under restricted feeding regimes with the use of RCs (36, 37). Subsequently, in studies offering cattle *ad libitum* access to both concentrate (38, 44) and forage-based diets (237), DMI and MY have been shown to be negatively correlated. As a result, the selection of animals on the basis of MY has the potential to increase the voluntary feed intake of future generations of livestock. However, the elevated feed intake associated with MY is unlikely to benefit animal performance, as a negative relationship of MY with measures of feed efficiency, such as residual feed intake (RFI; kg/day), has been revealed in studies implementing an *ad libitum* feeding regime (183, 237, 238). Therefore, a reduction in the DME associated with the selection of cattle for a low MY phenotype will likely occur at the expense of less efficient conversion of feed to animal protein, thus negatively impacting on-farm profitability. Therefore, the selection of animals on the basis of ratio traits should be avoided by virtue of their unpredictable response to other traits of economic importance in cattle production (18). Moreover, the effect of experimental conditions on the relationship between DMI and MY further highlights the complexities of CH_4_ output as a trait.

By virtue of its calculation, RME has been shown to be phenotypically and genetically independent of DMI and body weight in beef cattle but positively correlated with DME (36, 38). Recently, Smith et al. (143) highlighted beef cattle ranked as low for RME, produced 30% less CH_4_ (DME, MY, and MI), but had the same level of productivity (DMI, ADG, RFI, and carcass output) as high RME ranked animals. The lack of an apparent relationship of RME with traits of economic importance in cattle production makes it an attractive trait to utilize as part of the CH_4_ mitigation breeding program (239). Indeed, selection for a low RME phenotype has great potential to reduce the CH_4_ output of both future generations of breeding and finishing beef animals.

Indirectly selecting animals for enhanced feed efficiency has been advocated as an alternative selection index for reducing the CH_4_ output of livestock (10). Based on the literature, animals with an enhanced level of feed efficiency have been shown to produce less CH_4_ in some studies (101, 240–245). However, inconsistencies in the relationship between RFI and enteric emissions have been reported, with no difference in DME observed in animals with superior feed efficiency in some studies (183, 238, 246, 247).

As discussed, reducing days to slaughter is an effective CH_4_ mitigation strategy for beef cattle. However, slaughtering beef animals at an early age could have negative ramifications on both carcass output and quality. Berry et al. (248) proposed the trait, deviation in age at slaughter (DAGE), as an effective means of selecting animals with the enhanced genetic potential to reach slaughter weight over a shorter time frame, but produce a carcass of sufficient weight and fat cover to meet market specifications. Using records from over 2 million Irish cattle, DAGE was calculated from the regression of age at slaughter on carcass weight and fat score and was shown to have a heritability of 0.23–0.26 and both genetic and phenotypic standard deviation of 14.2–15.1 and 44.2 days, respectively. While the genetic potential exists to breed for faster-growing in-spec cattle, further in-depth analysis is needed to quantify the relationship between DAGE and CH_4_ production to fully verify the mitigation potential of breeding for a reduced age at slaughter.

## Conclusion

With an estimated 37% of enteric CH_4_ produced from grazing livestock (249), mitigation strategies that can be applied at pasture are urgently needed. Indeed for beef cattle, strategies requiring regular interaction with animals are not feasible during the grazing season, albeit some could have merit when animals are housed for winter. However, due to the low profitability of beef production systems (15), a winter CH_4_ supplementation strategy would at minimum need to be cost-neutral to ensure uptake at the farm level. Equally, altering the composition of the grazing swards to reduce CH_4_ emissions could be a challenge for extensive production systems due to the lack of long-term persistency of some alternative forages within the grazing sward (147) and complications with herbicide treatment. Therefore, in the absence of an effective antimethanogenic vaccine, the genetic selection of low-CH_4_-emitting animals is the only abatement strategy, which is likely effective for extensive pasture-based ruminant production systems. Nonetheless, breeding low-CH_4_-emitting ruminants will still be a challenge due to the large numbers of animals required to develop a breeding program (250), difficulties in performance testing cattle at pasture, and low uptake of reproductive technologies in non-dairy production systems (251).

Recently, the IPCC identified the urgent need to reduce the atmospheric concentration of CH_4_ to limit global temperature increases (4), with the EU, United States, and other countries pledging to reduce anthropogenic CH_4_ emissions by 30% by 2030 as part of the Global CH_4_ Pledge. However, reducing global enteric CH_4_ emissions will be a challenge due to the increase in animal production required to meet the projected demand for animal-sourced protein. Due to the linear relationship between DME and DMI, as reported by numerous authors (44, 143, 214), any CH_4_ mitigation strategies that support a higher level of feed intake have the potential to increase DME. For finishing beef cattle, any increase in DME as a result of higher feed intakes could potentially be offset by improved growth rates due to the relationship between DMI and average daily gain (252), leading to a reduction in the lifetime emissions of an animal. However, for the beef breeding herd, strategies that reduce DME will be required to meet international GHG reduction commitments. To fully understand the overall effects of different CH_4_ abatement strategies, future LCAs will need to be conducted to evaluate the effects of each CH_4_ mitigation strategy on total farm gate GHG emissions.

## Author contributions

All authors listed have made a substantial, direct, and intellectual contribution to the work and approved it for publication.
